# A Systematic Approach to the Design and Characterization of a Smart Insole for Detecting Vertical Ground Reaction Force (vGRF) in Gait Analysis

**DOI:** 10.3390/s20040957

**Published:** 2020-02-11

**Authors:** Anas M. Tahir, Muhammad E. H. Chowdhury, Amith Khandakar, Sara Al-Hamouz, Merna Abdalla, Sara Awadallah, Mamun Bin Ibne Reaz, Nasser Al-Emadi

**Affiliations:** 1 Department of Electrical Engineering, Qatar University, Doha 2713, Qatar; a.tahir@qu.edu.qa (A.M.T.); mchowdhury@qu.edu.qa (M.E.H.C.); amitk@qu.edu.qa (A.K.); sa1507714@student.qu.edu.qa (S.A.-H.); ma1508307@student.qu.edu.qa (M.A.); sa1509524@student.qu.edu.qa (S.A.); 2 Department of Electrical, Electronic & Systems Engineering, Universiti Kebangsaan Malaysia, Bangi, Selangor 43600, Malaysia

**Keywords:** gait analysis, characterization, smart insole, vertical ground reaction forces, force sensitive resistors, piezoelectric sensors, sensor calibration

## Abstract

Gait analysis is a systematic study of human locomotion, which can be utilized in various applications, such as rehabilitation, clinical diagnostics and sports activities. The various limitations such as cost, non-portability, long setup time, post-processing time etc., of the current gait analysis techniques have made them unfeasible for individual use. This led to an increase in research interest in developing smart insoles where wearable sensors can be employed to detect vertical ground reaction forces (vGRF) and other gait variables. Smart insoles are flexible, portable and comfortable for gait analysis, and can monitor plantar pressure frequently through embedded sensors that convert the applied pressure to an electrical signal that can be displayed and analyzed further. Several research teams are still working to improve the insoles’ features such as size, sensitivity of insoles sensors, durability, and the intelligence of insoles to monitor and control subjects’ gait by detecting various complications providing recommendation to enhance walking performance. Even though systematic sensor calibration approaches have been followed by different teams to calibrate insoles’ sensor, expensive calibration devices were used for calibration such as universal testing machines or infrared motion capture cameras equipped in motion analysis labs. This paper provides a systematic design and characterization procedure for three different pressure sensors: force-sensitive resistors (FSRs), ceramic piezoelectric sensors, and flexible piezoelectric sensors that can be used for detecting vGRF using a smart insole. A simple calibration method based on a load cell is presented as an alternative to the expensive calibration techniques. In addition, to evaluate the performance of the different sensors as a component for the smart insole, the acquired vGRF from different insoles were used to compare them. The results showed that the FSR is the most effective sensor among the three sensors for smart insole applications, whereas the piezoelectric sensors can be utilized in detecting the start and end of the gait cycle. This study will be useful for any research group in replicating the design of a customized smart insole for gait analysis.

## 1. Introduction

Gait analysis offers an opportunity for assessment of the act of walking, one of the most important features of the individual’s use pattern that displays posture in action. By identifying gait kinetics, gait kinematics and musculoskeletal activity, gait analysis can be utilized in various applications, such as rehabilitation, clinical diagnostics and sport activities [1]. Gait kinetics studies the forces and moments that results in movement of lower extremities during gait cycle. Vertical ground reaction forces (vGRFs) are the forces between the foot and ground which can be obtained by wearable sensors [2] and are considered as the main measurement in kinetic analysis. Gait kinetics have recently become a convenient tool for biomedical research and clinical practice. Different research teams studied the ability to diagnose or early detection of various diseases using gait analysis [3,4,5]. Some research teams used gait analysis in fall detection of elderly people, one of the most common domestic accidents among the elderly. With smart insoles, the fall event can be detected and doctors or personal who takes care of the elderly can be notified to take action. In athletic sports where walking, running, jumping and throwing are involved, gait analysis can be utilized to recognize an athlete’s faulty movement and, accordingly, enhance it. In addition, gait analysis can play positive role in the rehabilitation process for several diseases and complications.

Recently, with the development in sensor technologies, gait analysis using wearable systems became an effective approach [6,7,8]. Various types of wearable sensors such as force sensors, strain gauges, magneto-resistive sensors, accelerometers, gyroscopes, inclinometers etc. can analyze different gait characteristics. Accelerometers were used to conduct gait analysis studies, in which they were attached to feet or legs to measure the acceleration or velocity of human lateral movements during gait cycles [4]. Gyroscopes were used in gait analysis to measure the changes in orientation of lower body extremes with respect to the vertical axis. Goniometers measured the relative rotational motion between different body segments [2]. Electromagnetic tracking systems were developed as 3D measurement device that can be applied in the kinematic study of body movements [9].

Gait analysis is typically carried out using a force plate system or multi-camera-based system to capture the ground reaction forces (GRF) during different gait cycles. However, this method requires a costly set up and long post-processing time and can measure only limited number of strides. Therefore, it is not affordable by individuals for personal use [3,8,10]. Instrumented trade mills with few force plates laid on the trade mill are used by different research groups to mitigate the limitations of conventional force plates [2], but with treadmills restrictions are still present as subjects need to walk in a straight line where direction changes and turning cannot be realized. This led to an increase in research interest towards developing smart insoles, where wearable sensors can be employed to detect vGRF, joint movements, acceleration of lower extremities, and other gait variables [3,4,11,12]. vGRF is a useful tool to assess the health conditions of the patient, to enhance the performance of athletes [13,14,15]. Among different solutions for vGRF measurement, smart insoles have several extra advantages over force plates and multi-camera systems. Although force plates can measure shear forces and pressure changes, smart insoles are portable and capable of tracking motions and measuring pressure without rigid mounting, whereas the camera-based system requires large space for set-up along with long post-processing time. The smart insole offers flexible, portable, and comfortable solution for vGRF measurement. It is designed to monitor, process and display plantar pressure using pressure sensors embedded in the insole [3,4,11,12]. Recently, several off-the-shelf smart insoles have been offered by some companies (e.g., F-scan [16], MoveSole [17], Bonbouton [18], FeetMe [19] etc.), however, the commercial systems are very expensive for individual use, making it difficult for a home setting.

The aim of this study is to design and characterize smart insoles to detect vGRF during gait, with three different types of low-cost commercial force sensor: force-sensitive resistors (FSRs) [20], ceramic piezoelectric sensors [21], and flexible piezoelectric sensors [22]. All three types of sensor were calibrated before checking their suitability for smart insole application. A simple low-cost calibration method based on load cells is presented, mitigating the need to use expensive calibration devices or Motion Analysis Labs as a calibration reference. This work provides a systematic approach for sensor calibration guides, which can be replicated easily by other researchers to perform studies on smart insoles or other body-sensing technologies. To the best of our knowledge, this is the first article to compare three different low-cost commercially available force sensors for smart insole application.

The remainder of the article is organized into five sections. In Section 2, a comprehensive review of the recent works with smart insoles to detect vGRF in gait cycles are summarized. In Section 3, the experimental details for sensors calibration and insole characterization are presented. In Section 4, the mathematical analysis of each insole characterization and sensor calibration are explained. Results and a discussion are presented in Section 5. Finally, we conclude with future recommendations in Section 6.

## 2. Literature Review

Several research teams focused on fabricating and synthesizing the sensing parts or sensing fabrics of the smart insoles [23,24,25]. Sensing fabrics are fibers/yarns with sensing technologies or electrical components made of fabric materials, offering a flexible alternative to comfortably measuring human movement. Usually, piezoelectric, piezoresistive and piezo-capacitive materials are used to fabricate the sensing parts of the sensing fabrics, due to their elastic properties [26,27]. Shu et al., [26] implemented a low-cost insole with high pressure sensitivity using a fabric pressure sensing array made by the researchers with a pressure range of 10 Pa to 1000 kPa. It is attached to six locations corresponding to a polyimide film circuit board that takes the shape of the foot. They were able to measure the peak pressure, mean pressure, center of pressure (COP), and illustrate different pressure levels occurring at the six-targeted areas. However, the quality of the gait cycle records was poor, with irregular peak values, where the common gait shape with two peaks of the heel strike and toe off cannot be distinguished. Kessler et al., [27] demonstrated a low-cost flexible insole, made with Velostat and conductive ink electrodes printed on polyethylene terephthalate (PET) substrate. However, repeatability was a major problem and they proposed an averaging method to reduce the repeatability issue. However, the proposed method does not provide a generic solution for the force-sensing problem, it can be utilized only with periodic forces where spatial information is the key. On the other hand, some research teams used low-cost flexible force sensors to design the smart insoles [28,29,30] using commercially available piezoresistive [20], piezoelectric [21,22], capacitive transducers [2], fiber brag grating [5,31] sensors.

Piezoelectric force sensors are materials that generate electric charges when stressed. However, there are a few factors which limit the usage of piezoelectric sensors in smart insoles. The parasitic effect of piezo materials neutralizes the generated charge within a short time. Therefore, sophisticated electronics are needed to extract resultant charges, and this makes it difficult to use these sensors in measuring static or slow varying forces. In addition, protection circuits are needed, since piezo sensors generate high voltage values, which might reach above 100 V with peak vGRF values. Capacitive force sensors are another alternative force sensor, consisting of parallel capacitor plates that changes the capacitance in correspondence to applied force/weight. However, they need complex conditioning circuits and are highly subject to noise [20].

A commonly used body-sensing technology is the piezo-resistive sensor or FSR, which changes its conductivity based on the applied force. FSR is a polymer thick film (PTF) that is used to measure the applied force in different applications such as human touch and medical applications, industrial and robotics applications, and automotive electronics. The main advantages of FSRs are: thin size, very good shock resistance, low power requirement, fast response to force changes, robustness against noise, simple conditioning circuits, ability to fabricate using flexible materials, and low unit cost compared to other commercial force sensors [20]. However, these sensors have some disadvantages that need to be compensated for, such as non-linear behavior and repeatability error [3].

Bamberg et al. [4] used a combination of different FSRs, piezo electric sensors, accelerometers and gyroscopes to determine the vGRF. The main advantage of this approach is that it enables the detection of heel strike and toe off events in each gait cycle. In addition, it helps in estimating foot orientation and position. Even though gait variability can be analyzed by walking in a straight line, gait analysis concentrating merely on straight walking or running may not be adequate to interpret gait variability, since changing walking directions or turning have effects on extrinsic gait variability [11]. Similar research was done recently in [32], where the research group used the FSR sensor to develop the smart foot sole which transmits wirelessly the vGRF to a computer, and the patients were asked to walk on treadmill during the signal acquisition. Liu et al. [11] developed a wearable measuring insole using five triaxial force sensors in each shoe capable of measuring GRF and center of pressure (COP) on insole. The GRF results showed a great correspondence between the insole and the reference data. Kim et al. [33] conducted a similar study, where they have used similar triaxial force sensors and the sensors performance were tested on seven healthy male subjects. An in-shoe plantar measurement sensor with 64 sensing points made from an optoelectronics transducer covered with silicon in a matrix form covering 80% of contact region between the foot and the insole and handling capability of 1MPa was implemented by De Rossi et al. to measure COP and vGRF [5]. Howel et al. [3] demonstrated the design of a wearable smart insole using low-cost FSRs for gait analysis. This provided subject-specific linear regression models to determine the vGRF accurately using simultaneous collected data from motion analysis laboratory. However, insufficient information was given about the sensors calibration and the hardware design of the insole and the wireless system to transmit the data to host PC, making it difficult for other researchers to replicate the work.

Even though systematic sensor calibration with clear steps was followed by different research teams, expensive calibration devices were used to calibrate the force sensors. Some research teams carried out the experiments on the smart insoles in motion analysis labs, where simultaneous data collection from infrared motion capture cameras/RGB depth camera and force plates were done as reference measurement for the collected insole data [34,35]. In addition, some research teams used a universal testing machine to apply incremental weight values to sensor active area during calibration. Barnea et al., [36] used the CETR Universal Micro-Tribometer (UMT)-2 micro tribometer) device for calibrations, that can apply precise weights in X, Y and Z directions. Marco et al. [5] performed the sensor calibrations using robotic platform that can precisely apply controllable loads to the desired positions. Parmar et al., [37] evaluated the performance of 5 different commercial FSRs during static and dynamic loading with reliable test setups that can mimic realistic conditions when applying pressure on human limbs. The sensors were evaluated quantitatively based on their accuracy, drift, and repeatability behaviors. The tested sensors showed lower accuracy levels with static pressures compared to the dynamic pressure test, with high drift values. This necessitates the need for further study and analysis on the use of FSRs for static pressure applications.

## 3. Methodology

This section demonstrates the design of a complete system describing the main blocks of the smart insole along with illustrations of sensor calibration and insole characterization process.

### 3.1. Smart Insole Sub-System

**Figure 1:**
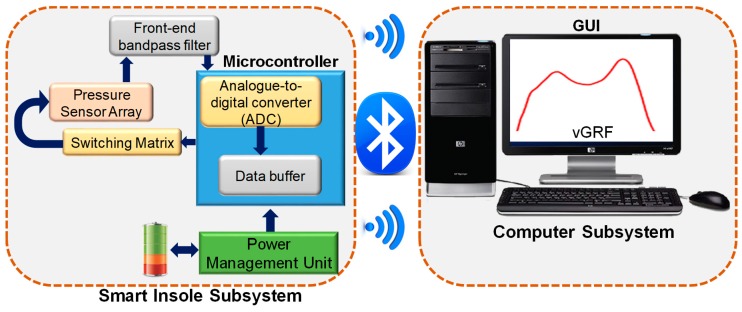
Smart insole block diagram.

Figure 1 shows the complete block diagram of the system, where the pressure sensor array was placed in a customized shoe above the control circuit. Pressure data were digitized through a microcontroller before they were sent wirelessly to a host computer for post processing and analysis. This subsystem was powered by a battery with the help of a power management unit. Pressure data were analyzed to extract various gait characteristics for different gait applications.

#### 3.1.1. Pressure-Sensing Array

The vGRF during gait cycles can be sensed using one of three alternatives:

A. Force-Sensitive Resistor (FSR)

**Figure 2:**
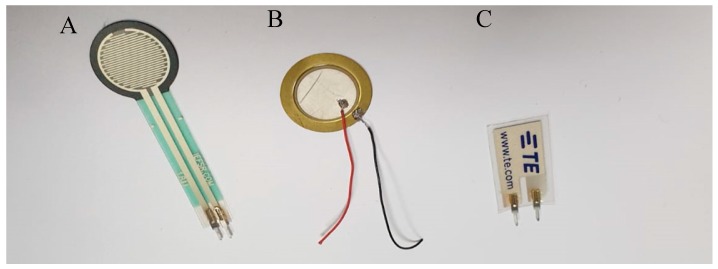
(**A**) Force-sensitive resistor (FSR) sensor from Interlink Electronics [20], (**B**) piezo-electric sensor from Murata Manufacturing Co. [38], (**C**) micro-electromechanical systems (MEMS) sensor LDT0-028K from Measurement Specialties Inc. [40].

The FSR exhibits a decrease in resistance as the applied force to the surface of the sensor increases. FSR sensors from Interlink Electronics [20] were used in this study as shown in Figure 2A. The sensors have a flexible round active area of diameter 12.7 mm to detect the applied force, with a two flexible lead wires to connect the sensor to the acquisition circuit. A FSR exhibits a non-linear relation between the applied force and the sensor’s resistance. In addition, no direct relationship is provided in the sensor’s datasheet. Therefore, proper calibration must be done prior to the sensor usage.

B. Ceramic Piezoelectric Sensor

A piezoelectric element is a sensor that produces an alternating voltage in response to an applied dynamic pressure or vibration. With applications related to dynamic forces, the piezoelectric sensor is highly recommended. When a force applied to the piezoelectric crystal element, the net movement of both positive and negative ions occurs. When there is a constant or zero pressure, the dipole is not formed [38]. It is important to mention that the force plate is originally made of piezoelectric material mounted between two metal plates to produce three-dimensional forces with a special mechanical arrangement [39]. This comes in different sizes; however, a ceramic piezoelectric element with 12.8 mm electrode diameter would be suitable to obtain a high-resolution pressure map as shown in Figure 2B.

C. Micro-Electromechanical Systems (MEMS) Sensor

The micro-electromechanical systems (MEMS) sensor is a new member of piezoelectric sensors family (Figure 2C). Similar to ceramic piezo electric sensors, it converts mechanical forces into electrical signals. However, the MEMS sensor can detect forces in x, y or z axes generating electrical impulses with positive or negative amplitudes depending on the force direction on a certain axis [40]. MEMS sensors are useful for detecting human motion sensor due to their flexibility, wide frequency range (0.001 Hz to 10 MHz), low acoustic impedance, high mechanical strengths, and high stability resisting moisture, etc. [40].

#### 3.1.2. Data Acquisition System

A. Microcontroller (MCU):

A microcontroller (MCU) was used to collect the data from the sensor and to send to the computer for classification. Simblee is a very compact and powerful ARM Cortex-M0 MCU with a six channels 10-bit analog-to-digital converter (ADC). It is featured with an inter-integrated circuit (I^2^C) and serial peripheral interface (SPI) communication interface, which were required for 9-degree of freedom (DOF) module. Moreover, it has an incorporated Bluetooth low energy (BLE) 4.0 module, which can be utilized to send data to the computer. This MCU operates on a power supply between +2.1 to 3.6 V.

B. Multiplexer (MUX)

Since MCU has a limited number of ADC channels whereas the number of sensors is needed for better spatial resolution of smart insole, it is suggested to use multiplexers (MUX) to reduce the number of required channels in MCU. A MUX allows several inputs in parallel to be routed into a single output depending on the input combinations of the data selectors. Active area of these sensors are close and sixteen sensors were used to create sensors’ array for each leg insole to obtain a high-resolution pressure map. Therefore, the CD74HC4067 multiplexer from Texas Instruments with 16 input channels was used in this study [41].

#### 3.1.3. Transmission Techniques

Three commonly used transmission techniques for connected biomedical sensors are ZigBee, Bluetooth Low Energy (BLE) and Wi-Fi. ZigBee is a two-way wireless communication technique developed for sensors and control networks, which need a wider range, low latency, low energy consumption at lower data rates. BLE is an alternative to the classical Bluetooth with higher data rate and low power consumption within a limited area with low latency at 2.4 GHz. Wi-Fi makes a good candidate for transmitting data with a data rate of up to 450 Mbps for indoor applications. However, it imposes latency on the system of more than 25 ms and higher power consumption. Table 1 shows a comparison between three different communication interfaces.

Since the smart insole was intended for indoor application, BLE and WiFi both were suitable for communication interface; however, the higher power consumption and latency made WiFi non-suitable for smart insole application. Moreover, Simblee MCU has in-built BLE in its small form factor. Therefore, BLE has been chosen as communication interface.

#### 3.1.4. Power Management Unit (PMU)

Power supplies were chosen depending on the operating voltage of the system components. The microcontroller and multiplexer both can operate at 3.3 V. The power management unit (PMU) is LiPo Charger/Booster module MCP73831 [47] and AMS1117 voltage regulator connected to a Lithium Polymer (LiPo) battery of 3.7 V (1000 mAh), which was regulated to 3.3 V. The PMU is not only delivering regulated 3.3 V to the system but also capable to charge LiPo battery.

#### 3.1.5. Host Computer

The acquired data from smart insole can be sent wirelessly to a host computer, where post processing, thereby displaying the vGRF as pressure maps during gait cycle, was carried out. The obtained data can be used in different gait analysis applications such as medical diagnostics, rehabilitation and athlete’s performance assessment.

### 3.2. Sensors’ Calibration

The first step in designing the smart insole is to calibrate the force sensors that are going to be used to detect the vGRF during the gait cycle. Three different force sensors were calibrated: FSR [20], piezo-electric sensor [21] and piezo -vibration sensor [22].

#### 3.2.1. Force-Sensitive Resistor (FSR) Calibration

**Figure 3:**
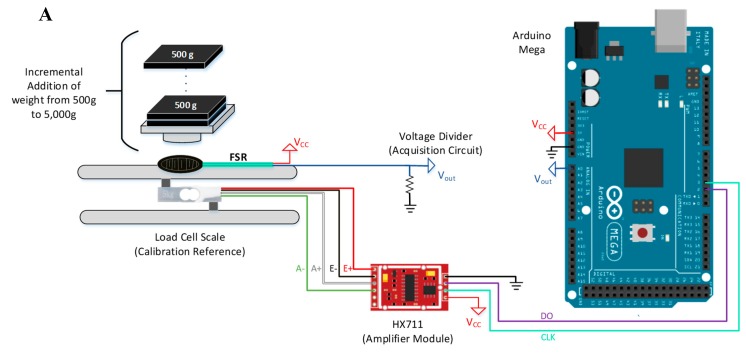

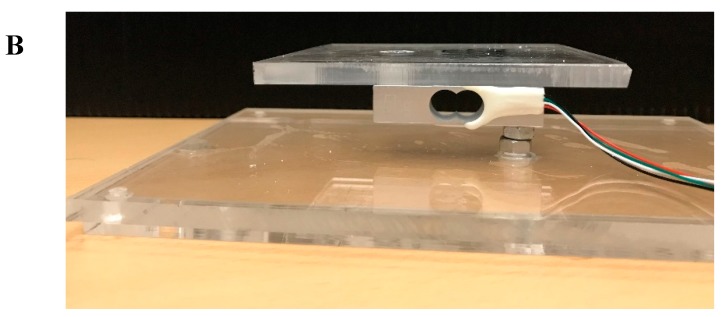
FSR calibration setup (**A**) and load-cell scale (**B**).

Firstly, a voltage divider circuit must be used with the sensor to convert the resistance change (due to applied force) of the sensor to a voltage value, which can be acquired by microcontrollers. Secondly, a load cell of 5kg from HT sensor technology company [48], with HX711 amplifier modules [15] was used as a weight reference for FSR calibration (Figure 3). The load cells consist of straight metal bar with two strain gauge sensors and two normal resistors arranged in a Whitestone bridge configuration, a constant excitation voltage (3–5 V) can be applied as an input to the circuit and the balanced configuration of the circuit replicates a zero output voltage in normal conditions when no force is applied. Any force applied to the load cell results in an unbalanced condition of the bridge leading to small voltage values in the output that can be detected and converted to force [48]. The load cell has high sensitivity and can detect as small as 1 gm of weight variation. However, the output voltage from the load cell is very small, with a maximum value of 5 mV. Therefore, a HX711 amplifier module was used. The amplifier module has instrumentational amplifier to amplify the signal with a 24-bit ADC that converts the analog signal from the load cell bridge to digital value that is readable by a microcontroller. The HX711 transmits data to the microcontroller using I^2^C communication protocol with 10Hz sampling rate [15].

The bar-type load cell was mounted with screws and spacers so that the strain can be measured correctly (refer Figure 3B). The load cell was placed between two plates with only one side screwed into each plate/board. This setup provides a moment of force on the strain gauges rather than just a single compression force, resulting in higher sensitivity to applied forces. The output voltage from the load cell exhibits a linear relationship with the applied force. This can be calibrated easily with any small object of known mass such as a coin that weighs a few grams.

A known weight object (ex. a coin) was placed on load cell plate; the calibration factor was adjusted until the output reading matches the known weight. Once the correct calibration factor is obtained, it was used to convert the load cell voltages to corresponding weights. The calibration factor is the slope of output voltage of load cell vs. real weights’ graph. The FSR was attached to adhesive material on the back face of the active area, which was used to fix the FSR on the scale. A cylindrical acrylic of 12.7 mm diameter, matching the active area of FSR, was used to apply force on the sensor only. In addition, a square shaped acrylic plate was glued on top of the cylindrical acrylic to support the weights, as shown in Figure 3. Then, 500 g weights are placed every 4 to 5 s until 5000 g is reached. Readings from load cell and FSR circuit are acquired simultaneously by Arduino, which were saved in a text file in a computer.

Finally, the FSR output voltage was plotted with respect to the load-cell weight and a mathematical relationship was derived. The equation was used to convert smarts insole FSR readings into the corresponding applied pressure by the foot.

#### 3.2.2. Piezo-Electric Sensor Calibration

**Figure 4:**
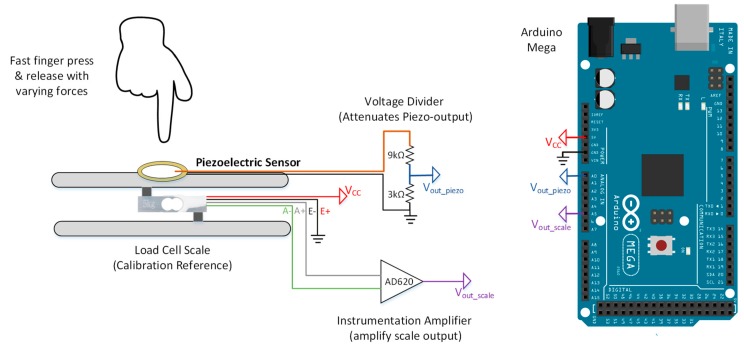
Piezoelectric sensor calibration setup.

The same load-cell module was used for piezo calibration with some modifications (as shown in Figure 4). Piezo transducers convert the applied mechanical forces into electrical impulses. Therefore, a high sampling frequency (above 50 Hz) is needed to acquire both the piezo output and the applied weights from the load cells. HX711 amplifier module samples the data with a low sampling frequency of 10 Hz. Therefore, the data was acquired directly by the 10-bit ADC of Arduino MCU with a sampling frequency of 1 kHz. However, AD620AN instrumentational amplifiers [39] were used before the acquisition step to amplify the small load cell outputs (maximum of 5 mV).

Firstly, a voltage divider circuit was used to reduce the high piezo voltage outputs, which can go up to 20 V. Secondly, the load cell was calibrated again due to the modification. Three dead weights of known masses were used: 500 kg, 2500 kg 5000 kg (maximum load for load cell). AD620AN instrumentational amplifier gain was adjusted to give an output of voltage when maximum load is applied. This ensures that the full range of the Arduino ADC was utilized. Three dead weights were added one by one on the scale and the output voltage from load cells were acquired by Arduino. A linear relationship was fitted between the load cell voltages and applied weights. This relationship was used to convert the load cell voltage to a corresponding weight.

Unlike the FSR, weights cannot be used to calibrate the piezo sensor, since piezo sensors are sensitive to dynamic forces only. Therefore, a fast finger press and release is suggested as an alternative. The calibration can be done by pressing the active area/ceramic of the sensor with various strengths and recording the generated electrical signals for each press as shown in Figure 4. Readings from the load cell and piezo voltage divider circuit were acquired simultaneously by the Arduino MCU. Serial terminal software was used to store the data in the computer. Load cell readings were plotted against the piezo output voltage and a linear relationship was derived. The equation was used to convert smarts insole readings into the corresponding applied weight by the foot.

#### 3.2.3. Micro-Electromechanical Systems (MEMS) Sensor Calibration

**Figure 5:**
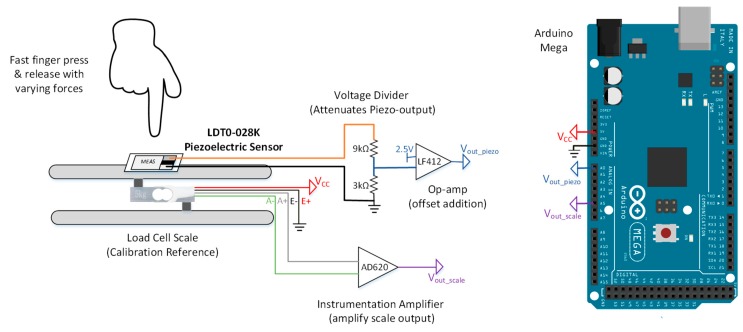
LDT0-028k MEMS sensor calibration setup.

MEMS sensors produce alternating current (AC) impulses with both positive and negative peaks. Therefore, little modification was required for the setup of piezo electric vibration sensor, refer to Figure 5. An offset circuit was added for the piezo-electric acquisition circuit. The piezo-vibration output voltage was reduced by a voltage divider circuit to ±1/2 V_cc_, then adder amplifier was used to add an offset of +1/2 V_cc_, so the new AC signal will be centered around +1/2 V_cc_ with maximum value of V_cc_ and minimum of 0 V. After modifying the acquisition circuit, the piezo electric sensors’ calibration steps were used to calibrate the piezo-vibration sensor.

### 3.3. Insole Fabrication

**Figure 6:**
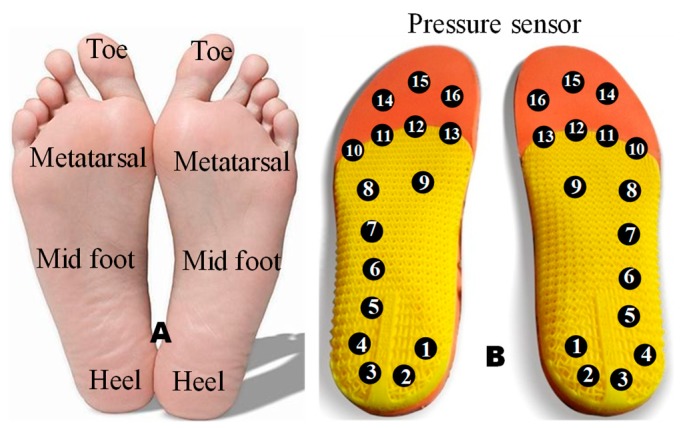
Area of foot selected for sensors (**A**), and array of pressure sensor (**B**) in those areas.

Once the sensors were calibrated, these sensors were separately used to construct the smart insole for vGRF detection during gait cycles. The FSR sensors and piezo-electric sensors were chosen to construct two different insoles. While the piezo-vibration sensor was found not suitable for vGRF detection, the reason of not selecting the piezo-vibration sensor is discussed in a later section. As shown in Figure 6, the most common place of the foot plane, where most of the pressure is exerted during gait are the heel, metatarsal heads, hallux and toe.

Sixteen sensors were placed on each insole to record pressure values in these areas. While no sensors were placed on the medial arch area of the foot as most people exert very low/no pressure on that area due to it is arch shape [49]. Smart insole data were collected from 16 FSRs/piezo-electric sensors. Sixteen inputs were multiplexed to one output through a 16-to-1 multiplexer and applied to an ADC input of the microcontroller then sent to host computer. All subjects were asked to place the sensor’s insole inside their shoes, then placing another layer of insole on top of it to ensure comfort of the subject while walking. The acquisition and transmission circuit were connected through a conductive pathway that can help in minimizing the size of the wire and avoiding any electrical hazard. The insoles were worn by the subject inside his/her own shoe while the acquisition and transmission circuits were placed inside a 7cm × 7cm box attached to the subject’s leg by an adhesive strap belt while acquiring the data. Acquired data were sent via Bluetooth to a computer, where they were plotted and analyzed.

#### 3.3.1. FSR Insole Characterization

**Figure 7:**
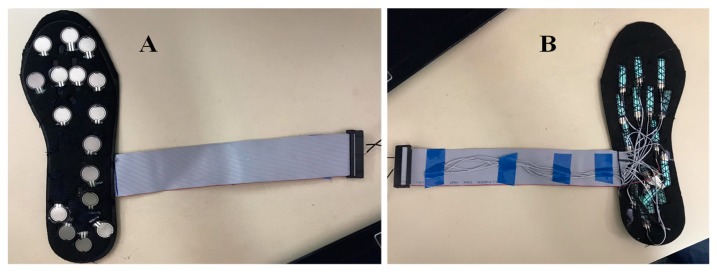
Smart Insole using FSR sensor: top (**A**) and bottom (**B**).

Twelve healthy subjects (Table 2) were asked to walk a straight 10 m walkway with self-selected cadence six times with an average walking speed of 3–4 mile per hour (MPH) and data acquired at 60 Hz sampling frequency using the smart insole made up of 16 FSRs (Figure 7). On treadmills, participants are restricted to walk in straight line as direction changes and turning cannot be realized; however, in the proposed study, the user walked freely in a 10-m walkway and they were asked to walk in a corridor which has a length of 10m and width of 1.5 m and they did not need to walk completely in a straight path and the user can walk in self-cadence, which is not possible on a treadmill. Subjects were asked to place the smart insole in their shoe while wearing cotton socks to avoid any sweat leakage that might damage the sensors or affect data acquisition from the sensors. Although walking speed is an important factor in some applications, it is not needed in many gait studies where the main focus is to detect the vertical ground reaction forces and asses the gait variables. The statistical gait variables were the symmetry between both feet, percentage of different phases (stance and swing phase) and sub phases (heel strike, mid-stance, toe off etc.) in a full gait cycle. Those statistical variables were used in various studies including sports or medical applications for gait analysis, without the need for walking speed measurement. However, the walking speed was recorded to see the impact of walking speed in the vGRF for a gait cycle. The FSR data were converted into force values by the relationship obtained in the calibration stage. Then 16 sensors’ data were added at each time instance to obtain one value that represents the full force exerted by the body while walking (i.e., vGRF).

#### 3.3.2. Piezo-Electric Insole Characterization

**Figure 8:**
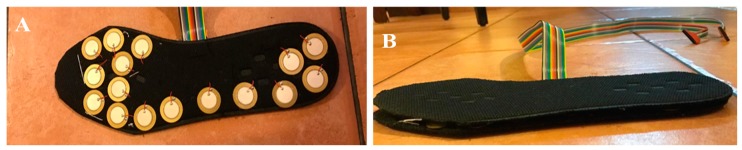
(**A**) Piezo insole with 16 piezo sensors, (**B**) additional insole layer placed on top on piezo insole to ensure comfortability.

A similar test was carried out with the piezo-electric sensor based smart insoles (Figure 8). Three subjects were asked to walk in a 10 m walkway in normal cadence, with three trials carried out by each subject. The data were acquired with a sampling frequency of 60 Hz.

### 3.4. Performance Evaluation of the Prototype System

**Figure 9:**
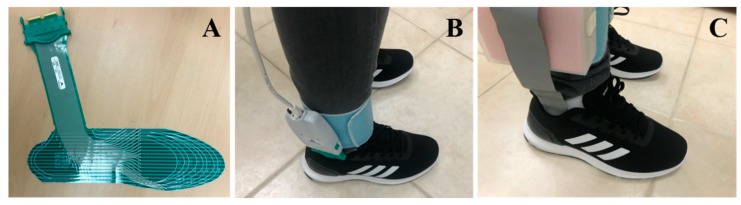
F-scan commercial system (**A**), F-scan system worn by Subject 01 (**B**) and FSR-based prototype system worn by Subject 01 (**C**).

A commercial F-scan smart insole system (Figure 9A) was used to validate the designed insole. The F-scan system is one of the best insoles currently available on the market. The insole comes with ultra-thin (0.18 mm) flexible printed circuit with 960 sensing nodes. Each sensing element was recorded with 8-bit resolution with a scanning speed up to 750 Hz. However, the overall cost of the system is 13,000 $ for the wired system and 17,000 $ for the wireless system. On the other hand, the instrumented insole costs only ~500 $. Usually, the vGRF peak is around ±10% of the subject’s weight. Therefore, using F-scan software, data collected from each subject was calibrated based on subject’s weight. The user needs to stand on one foot applying his/her full weight on the insole for 4 to 5 s, then the average applied weight was calculated. If the value obtained was less than the subject’s weight, the F-scan software adjusted the output by a multiplication factor. Similar approach was used in the prototyped FSR insole as well. Figure 9B,C show the F-scan and prototyped system worn by the same subject to compare the vGRF signal acquired by the individual system.

## 4. Analysis

This section explains the mathematical calculations and analyses used for the sensor calibrations and insole characterization.

### 4.1. Sensors’ Calibration

#### 4.1.1. FSR Sensor Calibration

The FSR sensors exhibits resistance change in correspondence to the applied force. Therefore, a voltage divider circuit was used to convert the resistance changes to voltage values to be acquired by microcontroller.

(1) $$V_{out}=V_{CC}\times \frac{R}{R+FSR}=5V\times \frac{11\;k\Omega \;}{11\;k\Omega +FSR}$$

As the applied force increases, the FSR resistance also decreases, showing an increased output voltage according to Equation (1). The acquired voltages were then converted to their equivalent FSR resistance values by substitution of Equation (1).

(2) $$FSR=\frac{5V\;\times \;11k\Omega }{V_{out\;}}-11k\Omega$$

#### 4.1.2. Piezo-Electric Sensor Calibration

The piezo-electric sensor generates high voltage values, as high as 20 V with weights less than 5 kg, which requires using a voltage divider circuit before data acquisition by microcontroller.

(3) $$V_{max\;input\;}=Voltage\;divider\;Gain\;\times V_{\mathrm{Piezo}\;\max}$$

(4) $$\Rightarrow Voltage\;divider\;Gain=\frac{{\;V}_{max\;input\;}}{V_{\mathrm{Piezo}\;\max}\;}=\frac{{\;V}_{CC}}{V_{\mathrm{Piezo}\;\max}\;}=\frac{5V\;}{20V}=0.25$$

Therefore, the voltage divider circuit were chosen as follows:

(5) $$V_{\mathrm{out}}=\frac{R1}{R1+R2}\times V_{\mathrm{Piezo}}=\frac{3\;M\Omega }{3\;M\Omega +9\;M\Omega }\times V_{\mathrm{Piezo}}=0.25V_{\mathrm{Piezo}}$$

Substituting the maximum piezo voltage in Equation (5) gives:

(6) $$V_{\mathrm{out}\;\max}=0.25V_{\mathrm{Piezo}\;\max}=0.25\left(20V\right)=5V$$

This ensures that maximum microcontroller input voltage (5 V) was not exceeded. The acquired voltages were then converted to their equivalent Piezo sensor voltage outputs by subject substitution of Equation (5).

(7) $$V_{\mathrm{Piezo}}=\frac{1}{0.25}\times V_{\mathrm{out}}=4V_{\mathrm{out}}$$

There are different equivalent electrical models for the piezo-electric sensors [28]. A simplified common model is a voltage source/generator with a capacitance, which was used in this study. Usually, the capacitance values are in Nano Farad range. The equivalent capacitance is typically measured using a parallel connection of a capacitance meter to the sensor. Connecting the piezo-electric sensor to the voltage divider circuit forms a first order high-pass filter. Therefore, high resistance values in mega ohms were used to ensure that most of the generated frequencies by the applied forces would pass. Assuming equivalent capacitance of piezo-electric sensor equal to 9 nF, the cut-off frequency can be written as:

(8) $$f_{cutt-off}=\frac{1}{2\pi RC}=\frac{1}{2\pi \left(3M+9M\right)9nF}=1.47Hz$$

Apart from DC and very low frequency components, other signal components were expected to be applied to the MCU input. AD620AN instrumentational amplifiers were used to amplify the low amplitude load cell signals, before it was applied to the microcontroller. The load cells give an output of maximum 40 mV, which can be amplified to the full-scale range of the analog channel. Therefore, the gain of the amplifier and the amplifier gain resistor were chosen as follows:

(9) $$G=\frac{V_{cc}}{V_{load\;max}}=\frac{5\;V}{40mV}=125$$

(10) $$R_{G}=\frac{49.9k\;}{G-1}=\frac{49.9k\Omega }{124}=402\;\Omega$$

#### 4.1.3. MEMS Sensor Calibration

As mentioned previously, the MEMS generates positive or negative amplitude signals based on the applied force in x, y or z directions. This requires an offset circuit along with a voltage divider circuit to reduce the signal amplitude. It is assumed the piezo-vibration output can go up to 10 V with the maximum applied force.

(11) $$V_{max\;input\;}=Voltage\;divider\;Gain\;\times V_{\mathrm{Piezo}\;\max}$$

(12) $$Voltage\;divider\;Gain=\frac{{\;V}_{max\;input\;}}{V_{\mathrm{Piezo}\;\max}}=\frac{V_{CC}/2}{V_{\mathrm{Piezo}\;\max}}=\frac{5V/2}{10V}=0.25$$

Therefore, the voltage divider circuit were chosen as follows:

(13) $$V_{\mathrm{out}}=\frac{R1}{R1+R2}\times V_{\mathrm{Piezo}}=\frac{3\;M\Omega }{3\;M\Omega +9\;M\Omega }\times V_{\mathrm{Piezo}}=0.25V_{\mathrm{Piezo}},$$

Substituting the maximum and minimum piezo voltage in Equation (13) gives:

(14) $$V_{\mathrm{out}\;\max/\min}=0.25V_{\mathrm{Piezo}\;\max/\min}=0.25\left(\pm 10V\right)=\pm 2.5V$$

The next step is to add an offset of 1/2Vcc to ensure that the signal was within 0 V to V_cc_ range.

### 4.2. Piezo-Electric Sensor Response

The piezo-electric sensors can detect the applied forces efficiently, by converting the mechanical movements into electrical signals. However, the movements need to be dynamic. The piezo-electric sensor generated electrical pulses that mimicked the applied mechanical movement. If the mechanical movement was a fast press and release of finger on the active area of the piezo-electric sensor, the pulse was shrunk to an impulse-liked shape.

**Figure 10:**
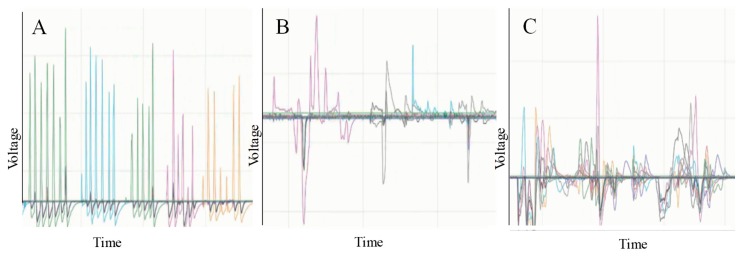
Piezo insole sensors output with Arduino serial plotter: (**A**) fast finger press and release, (**B**) slow palm press and release, (**C**) sensors output for two-step walking.

On the other hand, if a gentle force was applied by a slow press and remove by the palm of a hand, the generated signal had irregular pulse shape with longer duration compared to the fast finger press. Even though the piezo-electric sensor’s output can mimic dynamically changing force, it fails to detect a static force. Therefore, when the applied force is a mixture of dynamic and static force such as smart insole application, the piezo-electric sensors cannot be used to acquire static pressure. However, the piezo-electric sensors can be used to detect heel strike or toe off with good accuracy. Figure 10 illustrates the individual sensor output for the different applied forces.

**Figure 11:**
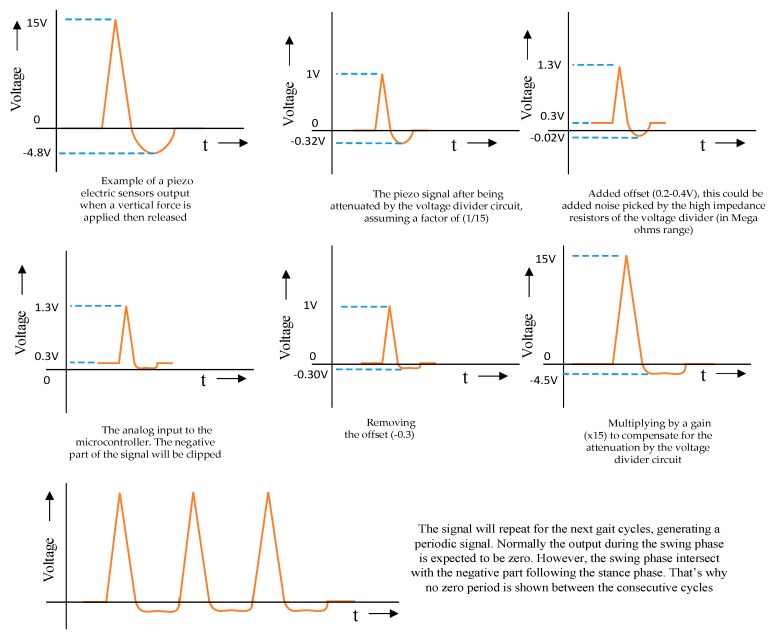
Mimicking piezoelectric sensor output during gait cycle.

Figure 11 shows the output from a single piezo-electric sensor of an insole for few gait cycles. When a force was applied vertically on the sensor’s active area (ceramic), it compressed, exerting an electrical impulse with a positive peak that mimicked the mechanical force applied. The electrical signal went back to zero. As the applied force was released, the signal continued to some negative values as the piezo ceramic bounced to the opposite direction of the applied force. Finally, the signal returned to zero. The microcontroller clipped the negative part of the signal. However, some part of the negative signal was still there, due to the offset added in the acquisition circuit as illustrated in Figure 11.

## 5. Results and Discussion

This section illustrates and discuss the results obtained from calibration and characterization tests.

### 5.1. Sensors’ Calibration

#### 5.1.1. FSR Sensor Calibration

**Figure 12:**
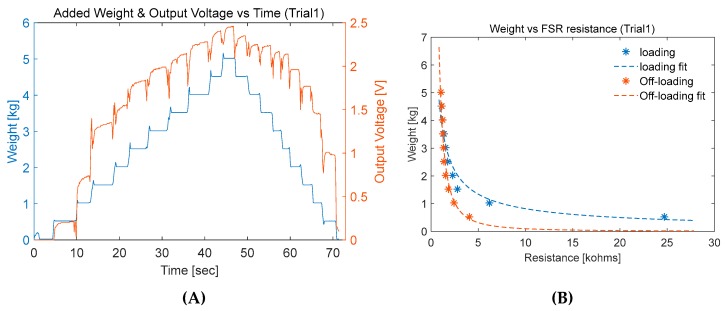
FSR calibration test (**A**) applied weight and FSR circuit output vs. time (**B**) applied weight vs. FSR resistance.

Three calibration trials were undertaken for one FSR sensor from Interlink Electronics [22], following the calibration procedure explained previously; 500 g weights where placed one by one every 3–4 s until it reached 5000 g, followed by unloading process from 5000 g down to 0 g. In the loading experiment, output voltage from the voltage divider circuit showed increasing values reflecting the decrease in FSR resistance as shown in Figure 12A. When the applied weight was constant, the output voltage remained constant with small variations.

In addition, if the constant weight was kept for a longer time (a few minutes), the sensor voltage stabilized to a steady value. However, the aim of this study was to investigate the dynamic response of the FSR. Therefore, the average output voltage for the sample were calculated and plotted against the corresponding applied weights. Figure 12B shows the plotted data with the fitted waveform. The calibration showed slight difference between the loading and unloading curves, which was expected due to the hysteresis behavior of FSRs. However, the error was caused by the FSR hysteresis, which can be neglected, as the difference was not significantly high. This can be justified if the response from the smart insole using FSR sensors resembles typical vGRF reported in the literature.

Off-Loading tests best fit relations:

$$Weight_{Trial1}=5035.2*Resistance^{-1.72}$$

$$Weight_{Trial2}=3436.5*Resistance^{-1.895}Weight_{Trial3}=8111.8*Resistance^{-2.589}$$

**Figure 13:**
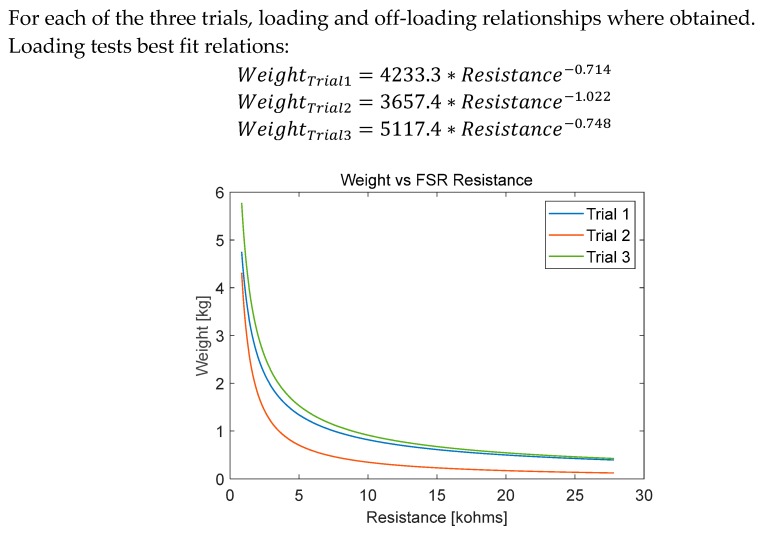
FSR calibration test: best fit curves between applied weight and FSR resistance for three trials.

It is evident that the first and third trial relationships were close to each other (Figure 13). Therefore, either of them can be chosen for the FSR insole. The second trial showed a steaper curve due to higher hystersis error.

#### 5.1.2. Piezo-Electric Sensor Calibration

**Figure 14:**
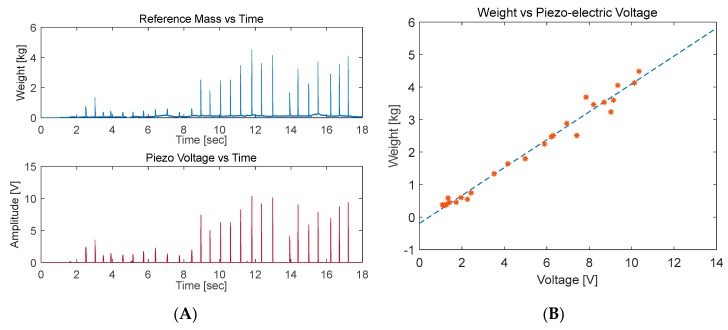
Piezo-electric sensor calibration test: (**A**) applied weight vs. time and piezoelectric output voltage vs. time (**B**) applied weight vs. piezoelectric output voltage.

Two piezoelectric sensors were used in the calibration process. Three trials were conducted on the first sensors with four trials for the second sensor. The piezoelectric sensors showed a linear relationship with the applied weights (Figure 14).

**Figure 15:**
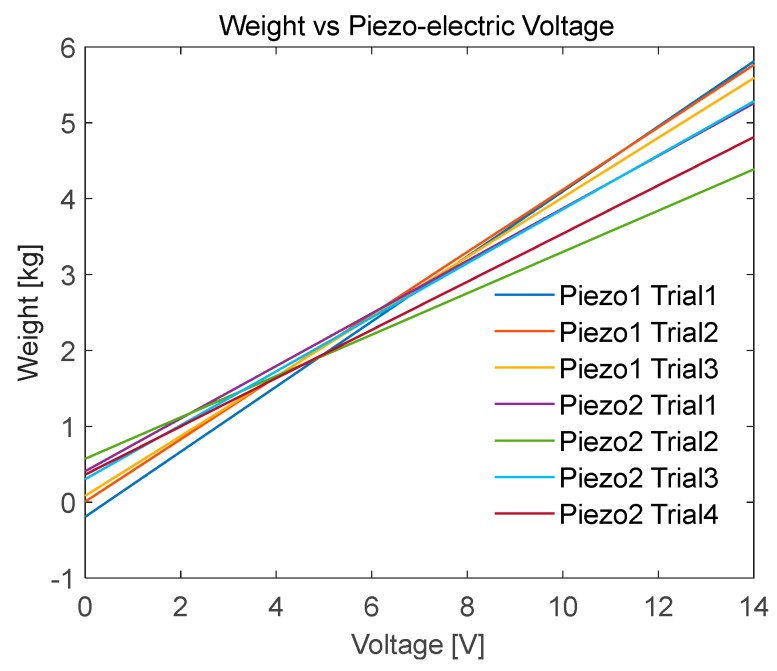
Piezoelectric sensor calibration test: seven trials shows relationship between the applied weight and piezoelectric output voltage.

The second and fourth trials for the 2nd piezo sensor had different slopes compared to the remaining trials. This could be related to the calibration process itself, as the weights were applied by fast presses and releases on the active area of the sensor. Therefore, applying the force on the exact same areas is not guaranteed between successive readings. This issue can be overcome by using a machine to apply the weights. However, this would defeat the purpose of the study in providing a low-cost setup (Figure 15).

Obtained lines of best fit:

$$Weight_{Piezo1Trial1}=0.42867*PiezoVoltage-0.19123$$

$$Weight_{Piezo1Trial2}=0.41110*PiezoVoltage+0.0081012$$

$$Weight_{Piezo1Trial3}=0.39321*PiezoVoltage+0.084656$$

$$Weight_{Piezo2Trial1}=0.34619*PiezoVoltage+0.4105$$

$$Weight_{Piezo2Trial2}=0.27242*PiezoVoltage+0.57351$$

$$Weight_{Piezo2Trial3}=0.35564*PiezoVoltage+0.30325$$

$$Weight_{Piezo2Trial4}=0.31765*PiezoVoltage+0.36416$$

#### 5.1.3. MEMS Sensor Calibration

Twenty different calibration trials were conducted on a piezo-vibration sensor. However, high repeatability error persisted, making it difficult to obtain a clear relation between sensor output voltage and the applied weight. The applied weight showed a direct proportional relation with output voltage for some successive readings and an inverse relation with some other successive readings. This is because of the MEMS sensitivity to the applied force in 3-D space (x, y or z directions). It generates 1-D output voltage with positive or negative amplitude depending on the applied force in certain direction. Therefore, if the applied force is a summation of forces in 2 or 3 axes, the output voltage might go to zero or attenuated with the addition of different sign amplitudes.

**Figure 16:**
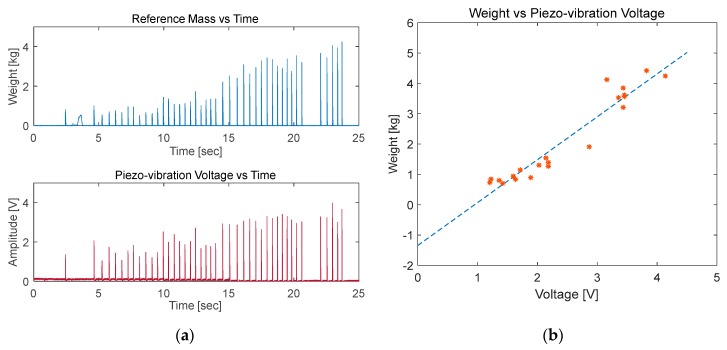
MEMS sensor calibration test: (**A**) applied weight vs. time and MEMS sensor output voltage vs. time (**B**) applied weight vs. MEMS sensor output voltage.

A linear relation was not clearly obtained by the application of vertical forces, as the applied force might not be applied in one axis only (Figure 16).

**Figure 17:**
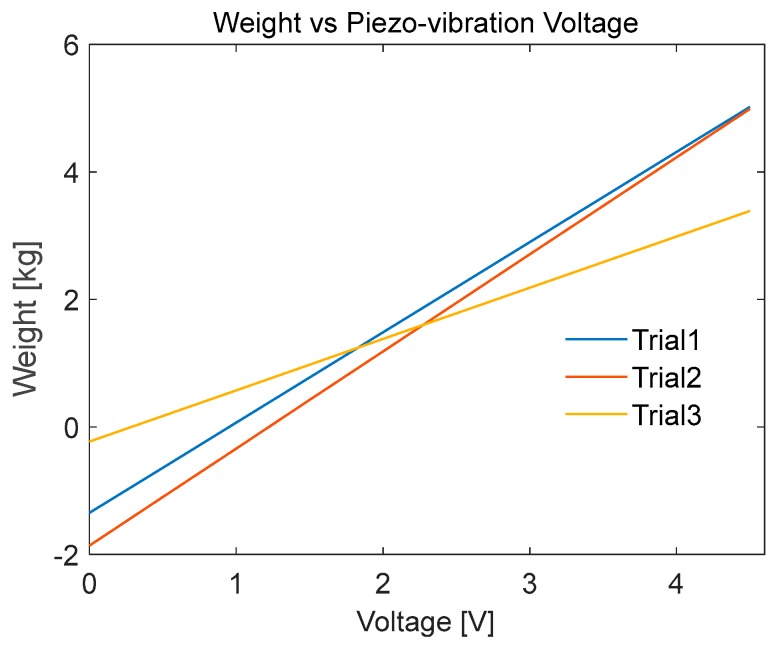
MEMS sensor calibration test: 3 best trials shows relationship between the applied weight and MEMS sensor’s output voltage.

The mathematical relations obtained in the calibration phase cannot be used to design a piezo-vibration sensor-based smart insole, since the applied force in gait can be in any of the x, y or z directions (Figure 17). Therefore, the piezo-vibration sensor was discarded from the sensor list for designing smart insole. However, it can be utilized in other biomedical applications where the force directions are limited to a certain axis or a fixed plane. Moreover, it can be used to detect initial timing of the applied force. The lines of best fit obtained were:

$$Weight_{Trial1}=1.4145*PiezoVoltage-1.3447$$

$$Weight_{Trial2}=1.5215*PiezoVoltage-\;1.8581$$

$$Weight_{Trial3}=0.80343*PiezoVoltage-\;0.22721$$

### 5.2. Insole Charecterization

#### 5.2.1. FSR-Based Insole Characterization

The gait cycle of 12 subjects were recorded while walking on a 10 m walkway in self-selected walking manner. Each subject had 6 trials recorded, where the first and last few (1 to 3) cycles were discarded from each trial, and the remaining part of the gait cycles for both feet were considered for analysis. The gait cycle of one of the subjects is analyzed in the following lines, illustrating a simple analysis technique that can be replicated in different application by researchers working with wearable insoles.

**Figure 18:**
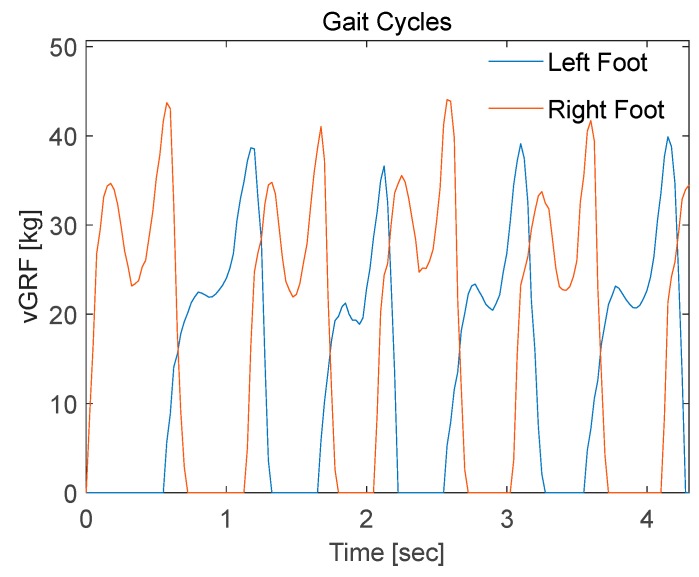
Gait cycles readings for left and right foot with FSR smart insole.

In normal gait cycles both heel peak (first peak) and toe off (peak) must show close values, with both feet having symmetrical signals. Even though the right foot signal showed close peak values (Figure 18), the left foot signals showed a big variance between the heel-strike and toe-off peaks. This can be explained, by the sensitivity difference between the FSRs of the insole and their hysteresis effect. As explained earlier, this issue was mitigated by some research teams using a regression models that calibrates the FSR insole readings against a reference signal, recorded simultaneously in motion analysis labs [34,35]. This expensive approach can be neglected in some applications, where the quality of the acquired signal is sufficient to achieve the desired goal. For instance, smart detection application, where the machine-learning algorithm can differentiate between different groups of people even with low- or medium-quality recorded gait cycles (vGRF).

The full gait record was segmented into distinct gait cycles. Then it was resampled into to 512 sample. Segmentation is a common practice to facilitate the comparison between all the gait cycles. The segmented gait cycles are used in smart detection algorithms where segments of equal length are used to train specific machine learning algorithms to classify different groups of people based on their gait. In addition, statistical data of the segmented cycles such as mean, standard deviation, time to peaks, and percentage of stance phase in a full cycle/stride (stance phase plus swing phase) can be utilized as a gait analysis tool in sports and medical applications.

**Figure 19:**
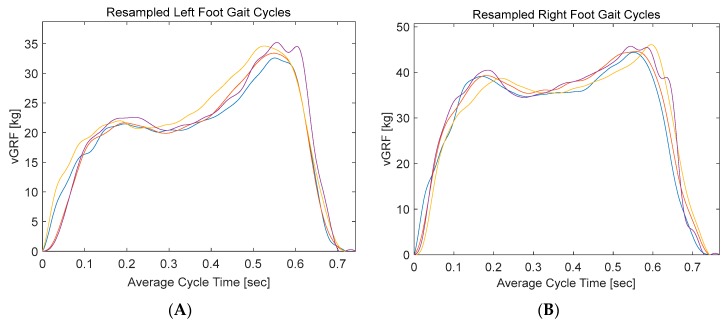
(**A**) Segmented left-foot gait cycles, (**B**) segmented right-foot cycles.

The segmentation was carried out by a customized MATLAB code that detects groups of consecutive non-zero samples. Then it segments those signals into individual stance phases, each starting with a heel-strike and ending with a toe off. Figure 18 shows the first 10-m trial of one of the participants, where four gait cycles were extracted after excluding the first and last two gait cycles. Left foot vGRF was segmented into four stance phases (Figure 19A).

**Figure 20:**
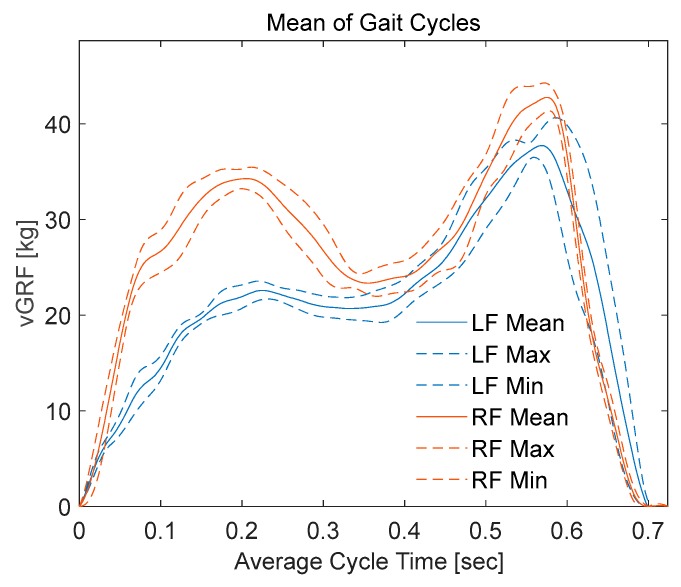
Means and standard deviations of gait cycles; blue curves represents the mean gait value of the left foot with dashed line representing the deviation from the mean, while orange curves represents the mean gait value of the right foot with dashed line representing the deviation from the mean value.

The data were sampled with a sampling rate of 60 samples/second, where each segment (stance phase) takes around 0.7 s. Therefore, each segment consists of around 42 samples, which were then resampled into 512 samples. The mean values and standard deviations of each of the 512 samples with respect to the 4 segmented signals were calculated. Then the mean values along with the deviation from the means (means plus and minus the deviation) for the left foot was calculated and plotted (Figure 20). Similar steps were repeated with left foot vGRF (Figure 20). This provides an illustrative figure that can be used by in different sport and medical applications to asses walking behaviors or complications.

The vGRF of a subject mainly depends on his/her health condition and the footwear used. In this study, all participants were advised to wear comfortable walking shoes avoiding high-heel shoes, especially for female subjects. This ensured that all subjects went through similar condition while conducting the experiment. It was observed that the collected data did not show any significant statistical difference based on gender.

#### 5.2.2. Piezoelectric Sensor Based-Insole Characterization

**Figure 21:**
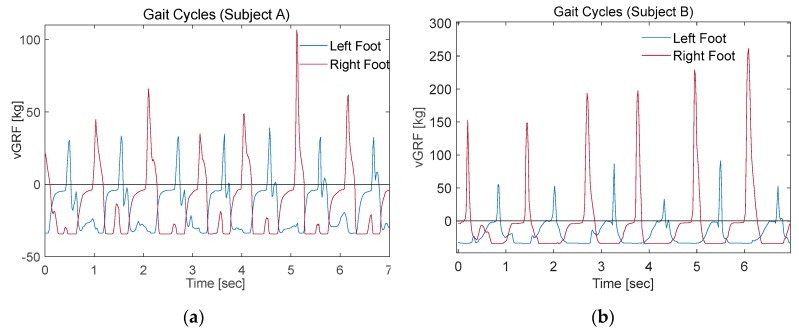
Gait cycles for left and right foot with piezoelectric smart insole (**a**) subject 1, (**b**) subject 2.

Three subjects participated in the piezo-electric insole test in the same manner as the testing of FSR based insole. The piezoelectric insoles were expected to detect the gait cycle, with impulse signals in heel-area sensors during the heel strike phase and lower amplitude impulses from all the sensors during the mid-stance phase. Finally, impulse signals from the toe and metatarsal heads sensors were taken in the toe-off phase. However, the readings were not promising, showing single irregular shape pulses per sensor for each individual gait cycle. The addition of different sensors output showed periodic impulses, one impulse per period (Figure 21). This indicates that the full stance period was detected as one event only. Meanwhile, the correct vGRF must show two distinct peaks between the mid-stance phase, summing up to three main phases: heel strike, mid stance and toe off. The rigid nature of the piezo sensor made it difficult to detect different gait phases. Therefore, it can be summarized that it is not suitable for the smart insole application which requires to produce reliable vGRF signal due to gait.

In this study, the authors have characterized three samples from each sensor category randomly; however, the smart insole was implemented using 16-sensors. Therefore, it was expected that there would be a small variation of the vGRF recorded from the smart insole in different trials and in different subjects. However, Figure 20 clearly depicts that the vGRF from an individual foot has a unique pattern and this finding matches with the vGRF recorded by the commercial smart insole and force plate. This reflects the fact that the smart insole designed using FSR is capable of acquiring vGRF reliably and the designed system is robust enough to adapt to the age, gender and body mass index (BMI) variation of the participants. However, carbon piezoresistive material (like Velostat), which authors have tested in preliminary experiments (not reported here in order to avoid unnecessary length of the manuscript), showed very high hysteresis and this type of material is not suitable for human dynamicity monitoring. On the other hand, piezoelectric sensors can monitor dynamic pressure variation however, they are very sensitive to small pressure change and incapable to reliably produce mean vGRF. Moreover, the vGRF changed over trials and over subjects significantly and, therefore, temporal feature of vGRF cannot be identified using piezoelectric sensor-based smart insole.

### 5.3. Performance Evaluation of the FSR-Based System

**Figure 22:**
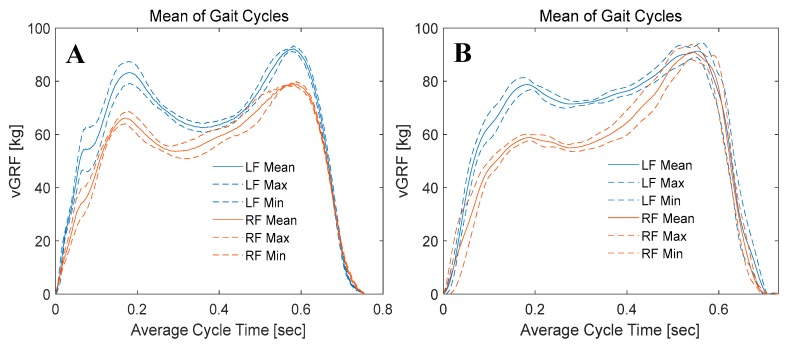
Comparison between the mean and standard deviation of vertical ground reaction forces (vGRF) from left (blue) and right (orange) foot using F-scan system (**A**) and FSR-system (**B**).

Comparing the mean and standard deviation of vGRF for a gait cycle of the same subject recorded using the two systems, commercial F-scan and the proposed FSR insoles, it can be seen that both showed good quality signals except slight differences in peak values (Figure 22). The FSR insole showed smaller vGRF during left-foot heel strike phase compared to the F-scan insole. This is an expected behavior as each sensor is somewhat unique due to the manufacturing process and we cannot calibrate individual sensors, which can lead to some variation. In addition, due to the presence of an insole, the sensitivity of some FSRs decreases more than the others in the shoe. To mitigate this problem, a highly uniform pressure should be applied across individual sensors. Each sensor should produce uniform output. When this is not the case for a specific sensor, the software should determine a unique scale factor to compensate for the output variation. Currently, there are a few companies such as T-scan, that provide a special piece of equipment (equilibration device) which applies a uniform pressure on the full insole using a thin flexible membrane to perform such calibration. Moreover, compared to the F-scan system, the FSR readings showed smaller differences between vGRF peaks and mid stance values. This was mainly due to the superior number of sensors for the F-scan system (960 sensing areas) compared to the proposed insole (16 FSRs). In addition, the F-scan sensing elements were uniformly distributed on the full foot area, while the FSR sensors were placed on the foot areas where most of the pressure is exerted with no sensors placed on the low-pressure areas (medial arch). Adding a few FSR sensors to the medial arch can improve the quality of the signal obtained, especially for subjects with flat foot, who exerts considerable amount of pressure on medial arch areas.

## 6. Conclusions

In this study, the authors have proposed and designed low-cost calibration setups for calibrating three different force sensors: FSR, ceramic piezoelectric and flexible piezoelectric sensors. The experiments conducted showed the effectiveness of the proposed setup in calibrating FSR and piezoelectric sensors, which are mainly affected by 1D force. It was found that the flexible piezoelectric sensors were performing poor in terms of calibration due to their sensitivity to 3D forces. Special force calibration machines are required to control the applied force in x, y or z directions. In addition, a systematic procedure for designing and characterizing two different smart insoles were illustrated. The vGRF signal acquired and segmented to obtain mean vGRF and its standard deviation for a gait cycle were calculated, which can be used to measure different statistical metrics (such as mean standard deviation, time to peak, etc.) that can help in assessing the walking behavior of athletes, patients or normal people. The FSR-based smart insole was able to acquire high quality vGRF for different gait cycles. On the other hand, the piezoelectric sensor-based insole failed to detect distinct gait phases. It cannot be utilized as an alternative to FSR in smart insole application. However, the calibrated piezo sensors can be utilized in other bio-sensing technologies such as detecting the start and end of each gait cycle.

## Figures and Tables

**Table 1:** Transmission methods comparison [42,43,44,45,46].

	Latency	Speed	Power Consumption	Range
**ZigBee**	15 ms	250 Kbps	9.3 mA	291 m
**Bluetooth Low Energy**	6 ms	1–11 Mbps	4.5 mA	10 m
**Wi-Fi**	≥25 ms	1.3 Gbps over 5 GHz and 450 Mbps over 2.4 GHz	35 mA	50 m

**Table 2:** Demographic variables of participants.

Number of Subjects	Age (Year)	Weight (kg)	Height (cm)	Body Mass Index (kg/m^2^)	Gender
7	30.1 ± 13.1	77.3 ± 21.2	159.8 ± 4.9	30.3 ± 7.9	Female
5	52.3 ± 4.8	83.5 ± 3.34	172.7 ± 11.7	28.3 ± 2.9	Male
